# Mincle activation enhances neutrophil migration and resistance to polymicrobial septic peritonitis

**DOI:** 10.1038/srep41106

**Published:** 2017-01-23

**Authors:** Wook-Bin Lee, Ji-Jing Yan, Ji-Seon Kang, Quanri Zhang, Won Young Choi, Lark Kyun Kim, Young-Joon Kim

**Affiliations:** 1 Department of Biochemistry, College of Life Science and Biotechnology, Yonsei University, Seoul 120-749, Republic of Korea; 2 Biomedical Research Institute, Seoul National University Hospital, Seoul 110-744, Republic of Korea; 3 Department of Integrated Omics for Biomedical Science, Graduate School, Yonsei University, Seoul 120-749, Republic of Korea; 4 Severance Biomedical Science Institute and BK21PLUS project to Medical Sciences, Gangnam Severance Hospital, Yonsei University College of Medicine, Seoul 06230, Republic of Korea; 5 Severance Institute for Vascular and Metabolic Research, Gangnam Severance Hospital, Yonsei University College of Medicine, Seoul 06230, Republic of Korea

## Abstract

Sepsis is a systemic inflammatory response to bacterial infection. The therapeutic options for treating sepsis are limited. Impaired neutrophil recruitment into the infection site is directly associated with severe sepsis, but the precise mechanism is unclear. Here, we show that Mincle plays a key role in neutrophil migration and resistance during polymicrobial sepsis. Mincle-deficient mice exhibited lower survival rates in experimental sepsis from cecal ligation and puncture and *Escherichia coli*–induced peritonitis. Mincle deficiency led to higher serum inflammatory cytokine levels and reduced bacterial clearance and neutrophil recruitment. Transcriptome analyses revealed that trehalose dimycolate, a Mincle ligand, reduced the expression of G protein–coupled receptor kinase 2 (GRK2) in neutrophils. Indeed, GRK2 expression was upregulated, but surface expression of the chemokine receptor CXCR2 was downregulated in blood neutrophils from Mincle-deficient mice with septic injury. Moreover, CXCL2-mediated adhesion, chemotactic responses, and F-actin polymerization were reduced in Mincle-deficient neutrophils. Finally, we found that fewer Mincle-deficient neutrophils infiltrated from the blood circulation into the peritoneal fluid in bacterial septic peritonitis compared with wild-type cells. Thus, our results indicate that Mincle plays an important role in neutrophil infiltration and suggest that Mincle signaling may provide a therapeutic target for treating sepsis.

Sepsis typically occurs when the initial host immune response is unable to contain the primary bacterial infection, and the condition can lead to systemic inflammation that culminates in multi-organ failure and death^1^,^2^. In sepsis, bacterial components such as lipopolysaccharide (LPS) induce the secretion of large amounts of proinflammatory cytokines from immune cells, often resulting in a lethal “cytokine storm”^2^. However, despite the presence of high levels of cytokines and chemokines, many cases of severe sepsis involve defects in the recruitment of immune effector cells into infection sites, resulting in a systemic impairment of innate immune function^3^,^4^. Therefore, elucidating the regulatory mechanism of immune cell recruitment is an important prerequisite for the development of therapeutic approaches for treating sepsis.

Neutrophils are an important component of the innate immune responses that constitute the first line of defense against bacterial infection. Neutrophils mediate the release of cytokines and chemokines, pathogen engulfment, and the generation of cytotoxic reactive oxygen species (ROS) and antimicrobial components^5^,^6^. Thus, aberrant neutrophil recruitment is frequently observed during various inflammatory disorders, including sepsis, trauma, and obesity, resulting in the failure to eliminate invading bacteria^3^,^7^,^8^,^9^.

Chemokine C-X-C motif ligand 1 (CXCL1) and CXCL2 are the major factors regulating neutrophil migration into infection sites^10^. Both CXCL1 and CXCL2 are recognized by the G protein–coupled receptor CXCR2, which mediates the reorganization of the actin cytoskeleton through activation of phosphoinositide 3-kinase (PI3K), mitogen-activated protein kinases (MAPK), phospholipase C (PLC), and Rho-GTPases^11^,^12^. However, prolonged ligand exposure leads to CXCR2 phosphorylation, resulting in receptor desensitization and internalization^13^. Moreover, activation of the toll-like receptors (TLRs) 2, 4, and 9 in conjunction with high levels of tumor necrosis factor-α (TNF-α) and nitric oxides results in downregulation of CXCR2 expression^14^,^15^,^16^,^17^,^18^. Therefore, high levels of cytokines could aggravate sepsis by reducing neutrophil migration via CXCR2 downregulation. Indeed, the recruitment of neutrophils to foci of infection is markedly reduced—along with reduced expression of CXCR2 on the surface of neutrophils—in sepsis patients^19^. Therefore, CXCR2 surface expression plays a critical role in modulating septic injury, but the underlying regulatory mechanism is poorly understood.

Along with TLRs, several C-type lectin receptors are known to play crucial roles in bacterial and virus infections^20^. In particular, stimulation with LPS or trehalose 6-dimycolate (TDM) induces high expression of Mincle (macrophage-inducible C-type lectin, also designated Clec4e or Clecsf9) on the surface of macrophages, dendritic cells, and neutrophils^21^,^22^,^23^,^24^,^25^,^26^. Mincle-deficient mice exhibit defective clearing of *Candida albicans*^27^ and reduced production of TNF-α and CXCL2 along with defects in neutrophil infiltration in response to *Malasezzia* fungal infection^26^. We previously showed that Mincle is required for mycobacterial cord factor–induced neutrophil recruitment to infection sites^24^. However, the mechanism by which Mincle regulates neutrophil migration is poorly understood.

Here, we provide evidence that Mincle promotes neutrophil recruitment by increasing CXCR2 surface expression in response to injury resulting from bacterial sepsis. Mincle-deficient mice exhibited an increased susceptibility to infection from severe cecal ligation and puncture (CLP; a model of acute polymicrobial septic peritonitis) and *E. coli–*induced septic peritonitis. In septic mice, fewer Mincle-deficient neutrophils infiltrated into the infection site due to reduced surface expression of CXCR2 and increased G protein–coupled receptor kinase 2 (GRK2) expression. Indeed, Mincle-deficient neutrophils did not exhibit CXCL2-dependent chemotaxis or F-actin polymerization. Therefore, in contrast to the repressive effect of TLRs, Mincle appears to regulate neutrophil migration by inducing CXCR2 surface expression via downregulation of GRK2 expression. Our data suggest that Mincle is an essential regulator of neutrophil activity in septic peritonitis.

## Results

### Mincle deficiency increases susceptibility to sepsis

**Figure 1:**
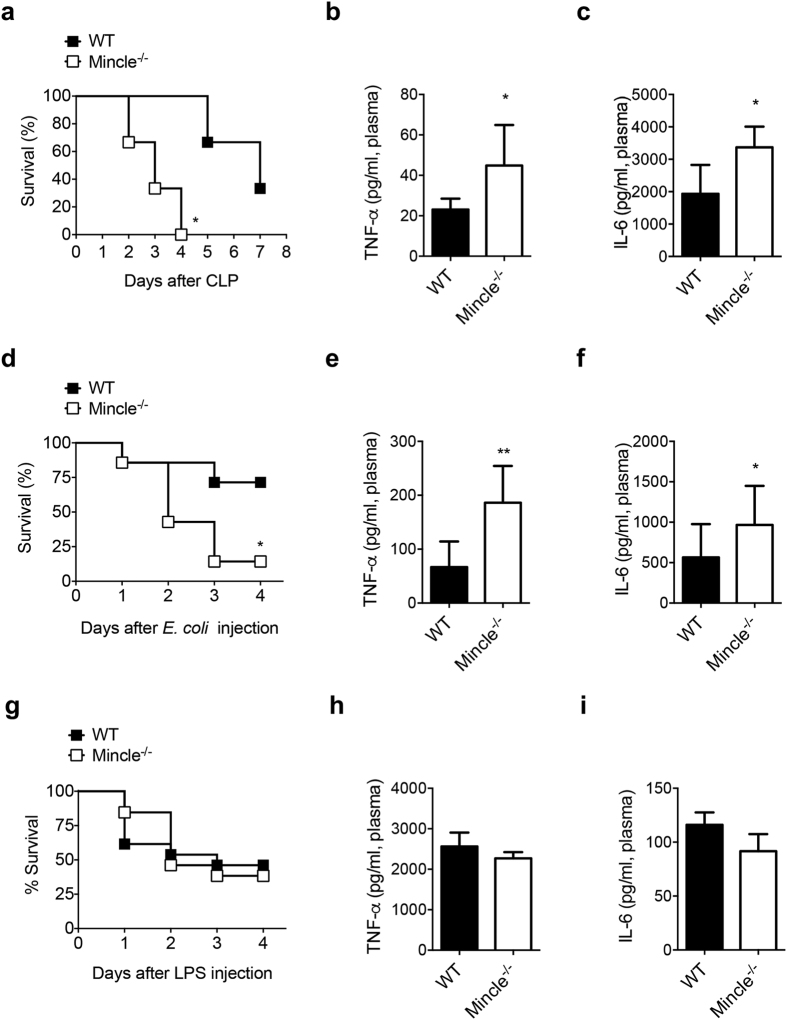
Mincle^−/−^ mice are a highly susceptible to CLP and *E. coli*–induced peritonitis but resistant to LPS-induced peritonitis. (**a**) Survival rate of WT and Mincle^−/−^ mice was determined daily up to 7 days after cecal ligation and puncture (CLP, n = 10). (**b**) TNF-α and (**c**) IL-6 levels in plasma of WT and Mincle^−/−^ mice (n ≥ 5 each) were measured 6 h after CLP. (**d**) Survival rate of WT and Mincle^−/−^ mice was determined daily up to 7 days after the onset of *E. coli*–induced peritonitis by intraperitoneal inoculation of bacteria (3 × 10^8^ CFU/mouse, n = 7). (**e**) TNF-α and (**f**) IL-6 levels in plasma of WT and Mincle^−/−^ mice (n ≥ 5 each) were measured 6 h after *E. coli* injection. (**g**) Survival rate of WT and Mincle^−/−^ mice was determined daily up to 7 days after LPS-induced endotoxemia by intraperitoneal injection (10 mg/kg, i.p. n = 13). (**h**) TNF-α and (**i**) IL-6 levels in plasma of WT and Mincle^−/−^ mice (n ≥ 5 each) were measured 6 h after LPS injection. Data are mean ± SD. *p < 0.05 relative to WT mice.

To explore the role of Mincle in polymicrobial sepsis, we induced septic peritonitis via CLP in wild-type (WT) and Mincle-deficient (Mincle^−/−^) mice. All Mincle^−/−^ mice succumbed within 4 d of septic challenge, whereas their WT littermates were resistant to CLP-induced polymicrobial sepsis for more than 7 d (Fig. 1a). As upregulated production of proinflammatory cytokines is the primary indicator of sepsis^28^,^29^, we examined the levels of TNF-α and interleukin (IL)-6 at 6 h after CLP (Fig. 1b,c). As expected, Mincle^−/−^ CLP mice exhibited higher plasma levels of TNF-α and IL-6 compared with WT mice. These data indicate that the high susceptibility to CLP-induced sepsis of Mincle^−/−^ mice correlates with increased production of systemic proinflammatory cytokines and chemokines.

As *E. coli* is one of the most commonly detected bacteria in both clinical sepsis in humans and experimental sepsis in mice, we investigated survival rates and proinflammatory cytokine production using a mouse model of *E. coli*–induced peritonitis. Similar to the CLP experiments, mortality was far higher among Mincle^−/−^ mice than WT mice following *E. coli* injection (Fig. 1d). Mincle^−/−^ mice also exhibited higher plasma levels of TNF-α and IL-6 compared with WT mice after *E. coli* injection (Fig. 1e,f). These data support the hypothesis that Mincle deficiency increases susceptibility to septicemia, with exaggerated production of proinflammatory cytokines.

The outer membrane of gram-negative bacteria, such as *E. coli*, is composed primarily of LPS, which is a critical factor in the pathogenesis of gram-negative sepsis^30^. We therefore, examined whether the increased susceptibility of Mincle^−/−^ mice to polymicrobial sepsis is due to responses to endotoxin. We administered a high dose of *E. coli* LPS (10 mg/kg) intraperitoneally and compared the effect on survival and cytokine production in both WT and Mincle^−/−^ mice. Contrary to the results of microbial sepsis experiments described above, no significant difference in survival rate was observed (Fig. 1g). Moreover, plasma TNF-α and IL-6 levels in Mincle^−/−^ mice at 6 h after LPS injection were similar to those of WT mice (Fig. 1h,i). These data demonstrate that Mincle does not play a major role in LPS-induced endotoxic shock.

### Mincle^−/−^ mice exhibit impaired bacterial clearance and reduced leukocyte recruitment in septic peritonitis

**Figure 2:**
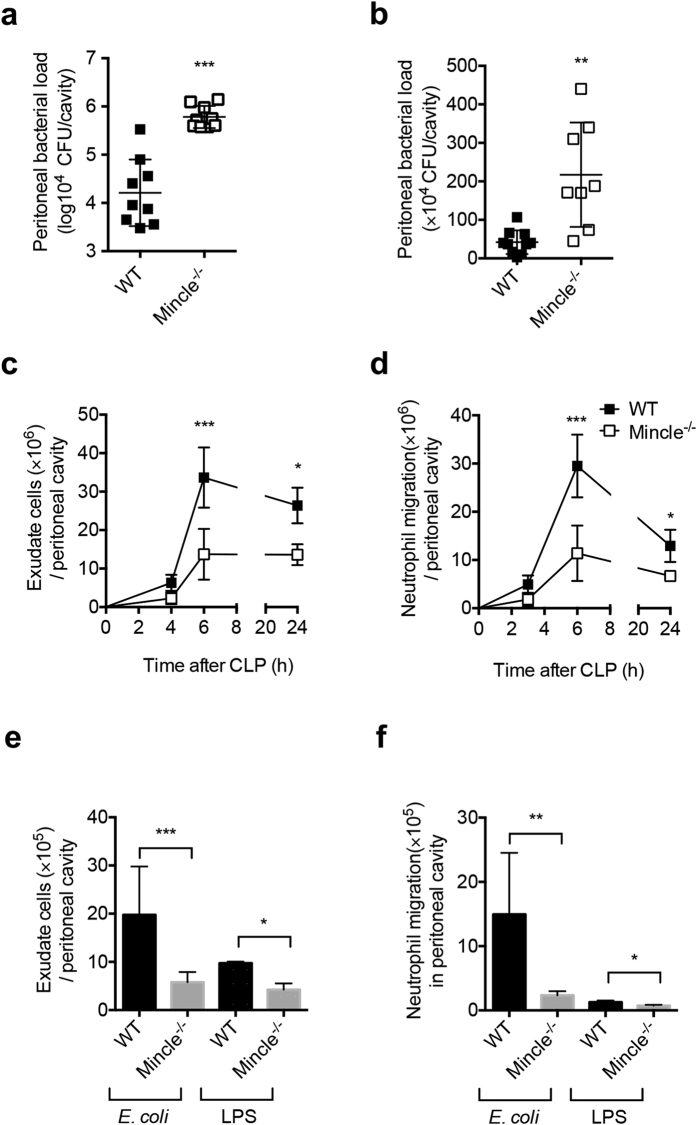
Mincle^−/−^ mice exhibit defective bacterial clearance and neutrophil migration after polymicrobial septic peritonitis and *E. coli*–induced peritonitis. (**a**,**b**) Number of CFU in peritoneal exudate 6 h after (a) CLP or (**b**) *E. coli* or LPS injection. Horizontal bars represent mean values, and squares represent individual mice (n = 8–10 each). (**c**) Total cells and (**d**) neutrophils in peritoneal exudate of WT and Mincle^−/−^ mice at the indicated time after CLP (n = 3–6 each). (**e**) Total cells and (**f**) neutrophils in peritoneal exudate of WT and Mincle^−/−^ mice 6 h after *E. coli* (n = 13) or LPS (n = 5) injection. Data are mean ± SD. *p < 0.05, **p < 0.01, ***p < 0.001 relative to WT mice.

Recognition of bacterial pathogens and induction of the host immune response to kill invading organisms are important steps in the resolution of septic inflammation^2^. Thus, we investigated the ability of WT and Mincle^−/−^ mice to recognize and kill invading bacteria and found that Mincle^−/−^ mice subjected to CLP and *E. coli–*induced peritonitis exhibited impaired clearance of bacteria in the peritoneum compared with WT mice (Fig. 2a,b). Because neutrophil recruitment is important for pathogen clearance during sepsis^9^, we examined the effect of Mincle deletion on immune cell recruitment after CLP and *E. coli*–induced peritonitis. Following CLP in WT mice, a substantial increase in leukocyte accumulation in the peritoneal cavity was observed, and neutrophils were the predominant cell type recruited (Fig. 2c,d). However, fewer total cells and neutrophils were observed in the peritoneum of Mincle^−/−^ CLP mice than WT CLP mice. Similarly, in Mincle^−/−^ mice with *E. coli*- or LPS-induced peritonitis, fewer infiltrating cells and neutrophils were observed in the peritoneum compared with WT mice (Fig. 2e,f). Although LPS injection in mice caused severe mortality and cytokine expressions as *E. coli* injection did, the level of neutrophil infiltration induced by LPS injection was lower compared to that induced by *E.coli* injection. Therefore, it is plausible that the endoseptic shock caused by LPS is mediated through cytokine storms rather than strong neutrophil infiltration. Nevertheless, neutrophil infiltration induced by LPS was reduced in Mincle^−/−^ mice compared to that in WT mice (Fig. 2f). Furthermore, the high susceptibility to CLP and *E. coli*–induced peritonitis of Mincle^−/−^ mice appears to be associated with impaired neutrophil recruitment and bacterial clearance.

To determine whether activation of Mincle signaling improves bacterial clearance and recruitment of leukocytes, we treated *E. coli*–induced peritonitis mice with the Mincle ligand TDM. TDM-treated mice showed enhanced bacterial clearance compared to a control group (Supplementary Fig. 1a). Moreover, more total cells and neutrophils were observed in the peritoneum of TDM-treated mice than the control mice (Supplementary Fig. 1b,c). Therefore, Mincle ligation seems to relieve *E. coli*–induced septic injury by enhancing neutrophil recruitment and bacterial clearance.

### Mincle regulates GRK2 expression

**Figure 3:**
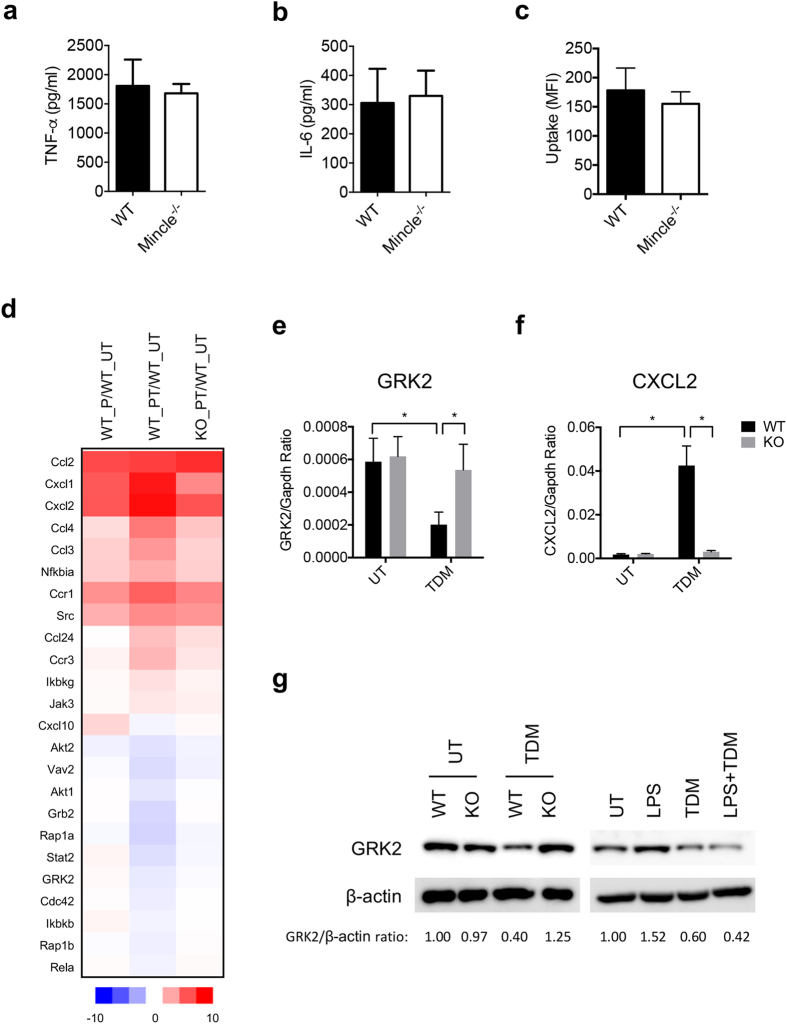
Mincle signaling downregulates GRK2 expression. (**a**,**b**) Neutrophils isolated from BM of WT and Mincle^−/−^ mice were infected with live *E. coli* (DH5α, MOI = 10). At 24 h after infection, (**a**) TNF-α and (**b**) IL-6 levels were determined by ELISA. (**c**) Neutrophils isolated from BM of WT and Mincle^−/−^ mice were incubated with green fluorescent protein–expressing *E. coli* in medium containing 10% normal fetal bovine serum for 1 h, after which phagocytosis was quantified using flow cytometry. (**d**) Heatmap distribution showing changes in chemokine signaling–related gene expression up- or downregulated by Pam3 and TDM co-stimulation (GSE70793). WT and Mincle^−/−^ bone barrow-derived macrophages (BMDMs) were stimulated with Pam3 (P) or co-stimulated with Pam3 and TDM (PT) for 12 h, after which RNA sequencing was performed. Heatmap showing log2 expression ratio to untreated (UT). WT_P/WT_UT; comparing Pam3 treated to untreated in WT, WT_PT/WT_UT; comparing Pam3 and TDM co-treated to untreated in WT, KO_PT/WT_UT; comparing Pam3 and TDM co-treated in KO to untreated in WT. (**e–f**) (**e**) Grk2 and (**f**) CXCL2 mRNA levels in WT and KO BM neutrophils stimulated with TDM for 12 h were analyzed by qRT-PCR. (**g**) GRK2 protein expression in WT and KO BM neutrophils stimulated with LPS and/or TDM for 24 h was analyzed by immunoblotting. *p < 0.05 relative to the indicated sample.

To investigate whether Mincle suppresses bacterial infection, we examined the production of TNF-α and IL-6 by BM neutrophils infected with live *E. coli*. However, the release of these cytokines was comparable between WT and Mincle^−/−^ neutrophils (Fig. 3a,b), indicating that Mincle does not appear to induce proinflammatory cytokine production in response to bacterial infection.

Phagocytosis plays a major role in eradicating bacterial infections. Following pathogen invasion, neutrophils move to the foci of infection, where they phagocytize the microbes and destroy them with ROS and granule enzymes or via the secretion of neutrophil extracellular traps (NETs)^31^. To investigate whether Mincle regulates the bactericidal activity of neutrophils, we assayed the phagocytosis of *E. coli* cells by WT and Mincle^−/−^ neutrophils. No differences in the phagocytosis of *E. coli* cells were observed, however (Fig. 3c), indicating that Mincle is not required for the phagocytosis of *E. coli*.

To elucidate the molecular signature associated with Mincle-dependent neutrophil recruitment, we examined changes in the transcription of factors related to chemokine signaling pathways using transcriptome analyses of WT and Mincle^−/−^ macrophages stimulated with the respective ligands. Interestingly, we found that the transcription of CXCL1 and CXCL2 was upregulated by TDM treatment, whereas transcription of GRK2 was downregulated (Fig. 3d). Rapid neutrophil recruitment to the site of infection is essential for restricting the spread of bacteria and facilitating their complete destruction. With respect to neutrophil infiltration, reduced CXCR2 expression on the membrane of circulating neutrophils is associated with a decline in neutrophil migration^16^. In addition, previous studies have demonstrated that GRK2 plays a prominent role in the phosphorylation and downregulation of chemokine receptors, such as CXCR2, in leukocytes^32^,^33^.

From our results, we postulated that Mincle signaling plays an important role in driving neutrophil migration by suppressing GRK2 expression, in turn blocking the downregulation of CXCR2. We, therefore, investigated TDM-induced expression of CXCL2 and GRK2 mRNA in isolated BM neutrophils using qRT-PCR. The results of qRT-PCR analyses agreed with the transcriptome analyses, demonstrating that TDM stimulation leads to a significant upregulation of CXCL2 and downregulation of GRK2 expression (Fig. 3e,f). Similarly, GRK2 protein expression was significantly downregulated in TDM-treated neutrophils compared with control cells (Fig. 3g). In addition, GRK2 is upregulated by LPS^34^,^35^, and promotes a process of CXCR2 desensitization^3^,^34^. These findings prompted us to examine the effect of TDM on LPS-induced expression of GRK2 in BM neutrophils. Indeed, GRK2 protein was further induced by LPS stimulation, but strongly suppressed by co-treatment with TDM (Fig. 3g). Of note, Greco *et al*. recently showed the downregulation of TLR4 signaling by Mincle^36^. Therefore, Mincle could reduce GRK2 expression in sepsis both directly and indirectly by downregulating TLR4 signaling.

### Mincle^−/−^ neutrophils exhibit downregulated surface expression of CXCR2 during bacterial sepsis

**Figure 4:**
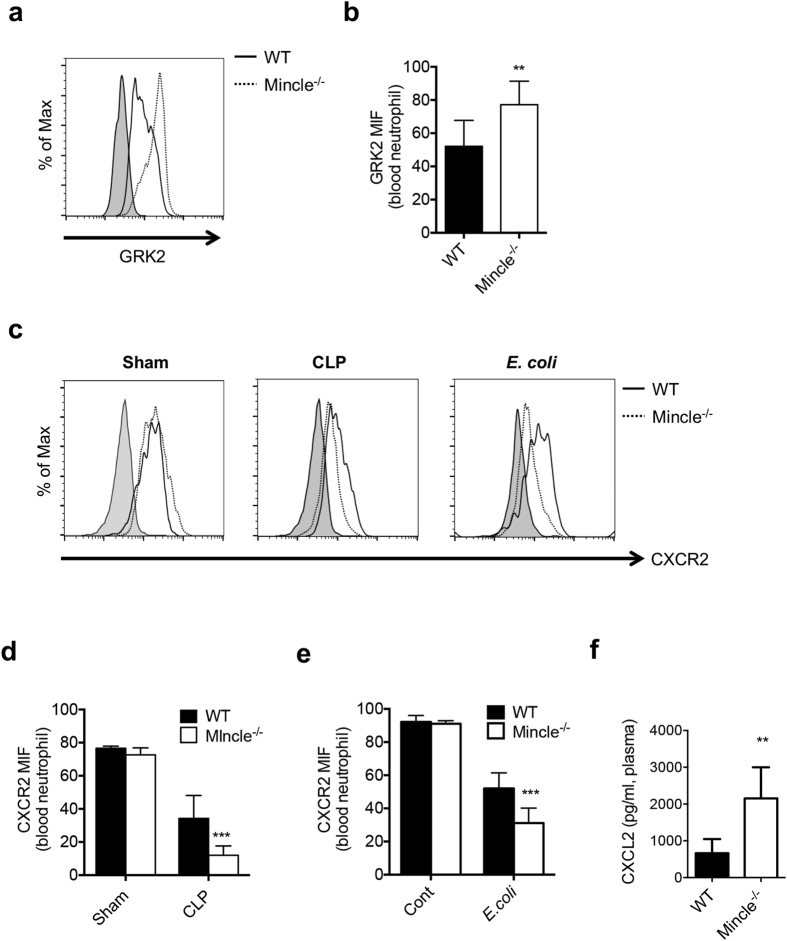
Mincle^−/−^ neutrophils exhibit reduced CXCR2 surface expression and upregulation of GRK2 expression compared with WT neutrophils during septic peritonitis. (**a**) GRK2 expression on blood neutrophils (Gr-1^+^ CD11b^+^) of WT and Mincle^−/−^ mice 2 h after CLP, measured using flow cytometry. (**b**) Average MFI from (**a**). (**c**) Surface expression of CXCR2 on blood neutrophils measured using flow cytometry. Neutrophils (Gr-1^+^ CD11b^+^) 2 h after sham (normal), CLP, or *E. coli*–injected WT and Mincle^−/−^ mice were measured. (**d**,**e**) Expression of CXCR2 by blood neutrophils from (**d**) CLP or (**e**) *E. coli*–injected WT and Mincle^−/−^ mice was quantified based on MFI. (**f**) CXCL2 level in plasma of WT and Mincle^−/−^ mice (n ≥ 5 each) was measured 6 h after CLP in WT and Mincle^−/−^ mice. *p < 0.05, **p < 0.01, ***p < 0.001 relative to WT CLP or *E. coli*–injected mice.

To investigate whether Mincle regulates GRK2 expression in CLP sepsis, we quantitatively examined GRK2 expression. The expression of GRK2 protein was significantly upregulated in the blood neutrophils of Mincle^−/−^ CLP mice (Fig. 4a and b). To evaluate the role of Mincle in the regulation of CXCR2 expression, we analyzed the surface expression of CXCR2 on neutrophils from septic mice. High and similar levels of CXCR2 surface expression were observed on neutrophils from both WT and Mincle^−/−^ sham mice (Fig. 4c,d). The level of CXCR2 expression on neutrophils from WT CLP mice was half of that on neutrophils from sham mice due to ligand-induced internalization and recycling. However, the level of CXCR2 on the surface of neutrophils from Mincle^−/−^ CLP mice was very low compared with neutrophils from control WT mice. In addition, neutrophils from Mincle^−/−^ *E. coli*–infected mice also expressed less CXCR2 than neutrophils from *E. coli*–infected WT mice (Fig. 4c,e and Supplementary Fig. 2). Mincle ligation by TDM treatment increased CXCR2 expression on neutrophils from *E. coli*-infected mice (Supplementary Fig. 1d). Although neutrophils from Mincle^−/−^ CLP mice showed decreased CXCR2 expression which correlated with their defective recruitment, the mutant mice exhibited higher levels of CXCL2 expression than did WT mice (Fig. 4f). These results most likely reflect the indirect consequences of diminished neutrophil recruitment in Mincle^−/−^ mice (which provokes additional chemokine responses) than normal Mincle-mediated neutrophil recruitment. Therefore, these data suggest that the decreased surface expression of CXCR2 on Mincle^−/−^ neutrophils is accompanied by reduced migration into the peritoneal fluid during bacterial septic peritonitis.

### Decreased CXCL2-induced chemotaxis in Mincle^−/−^ neutrophils

**Figure 5:**
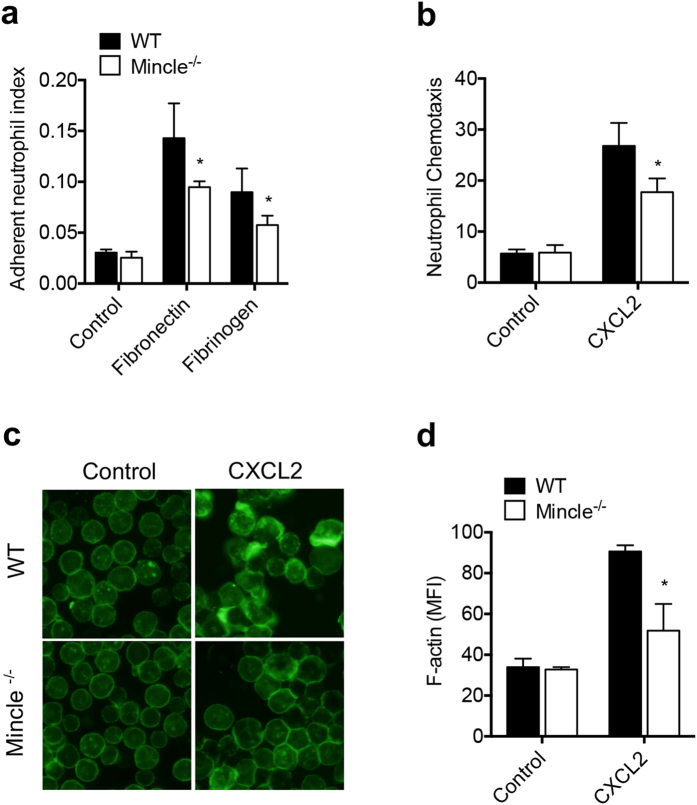
Mincle^−/−^ neutrophils exhibit reduced chemotactic activity. (**a**) CXCL2-induced adherent neutrophils. WT or Mincle^−/−^ BM neutrophils were allowed to adhere the fibronectin- or fibrinogen-coated plates for 4 min and then stimulated with 10 ng/ml CXCL2 for 30 min. (**b**) Chemotaxis of WT or Mincle^−/−^ BM neutrophils to 10 ng/ml CXCL2 for 30 min. (**c**) F-actin polymerization of WT or Mincle^−/−^ BM neutrophils after CXCL2 treatment. Representative images from three independent experiments are shown. (**d**) F-actin polymerization was quantified based on MFI. *p < 0.05 relative to the WT.

Based on our finding of Mincle-mediated CXCR2 regulation on neutrophils, we postulated that Mincle similarly inhibits CXCL2-induced neutrophil adhesion and migration. Neutrophil adhesion induced by CXCL2 was assessed using fibronectin- or fibrinogen-coated plates. Compared with WT neutrophils, Mincle^−/−^ neutrophils exhibited impaired adhesion to both fibronectin- and fibrinogen-coated plates (Fig. 5a).

We then examined *in vitro* chemotactic responses to CXCL2. Compared with controls, CXCL2 significantly enhanced the migration of WT neutrophils (Fig. 5b). However, the migration of Mincle^−/−^ neutrophils in response to the chemoattractant was impaired, compared with CXCL2-treated WT cells.

Cellular movement is mediated primarily by actin remodeling^37^. To investigate whether Mincle activates actin remodeling in CXCL2-induced neutrophil migration, we examined CXCL2-induced F-actin polymerization in WT and Mincle^−/−^ neutrophils. F-actin polymerization induced by CXCL2 increased significantly in WT neutrophils compared with Mincle^−/−^ neutrophils (Fig. 5c,d). These results, thus, demonstrate that Mincle is involved in CXCL2-mediated adhesion, chemotactic responses, and F-actin polymerization in neutrophils, and that the high susceptibility of Mincle^−/−^ mice to septic peritonitis can be attributed in part to these responses.

### Mincle^−/−^ neutrophils exhibit impaired migration into sites of infection during bacterial sepsis

**Figure 6:**
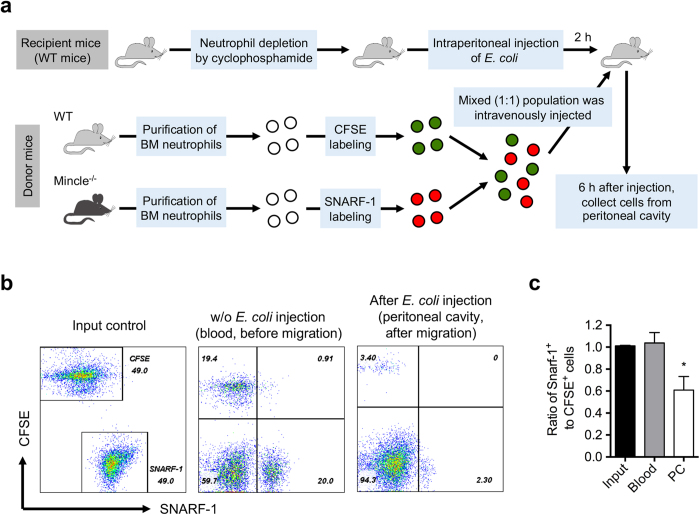
Mincle^−/−^ neutrophils migrate less efficiently into the peritoneal cavity after *E. coli–*induced peritonitis. (**a**–**c**) *Ex vivo* adoptive transfer of WT or Mincle-deficient neutrophils. (**a**) BM neutrophils of WT or Mincle^−/−^ mice were purified and labeled with the intracellular fluorescent dye CFSE or SNARF-1. Labeled cells were mixed (1:1) and infused i.v. into WT recipient mice challenged with *E. coli* injection. Peritoneal lavage was collected 6 h after cell injection, and the ratio of adoptively transplanted WT and Mincle^−/−^ neutrophils was determined using flow cytometry. (**b**) Representative flow cytometry results of input and recovered cells from the blood 2 h after i.v. of the mixed cells without *E. coli* injection for the control of before migration and from the peritoneal cavity 6 h after *E. coli* injection. (**c**) The ratio of adoptively transplanted Mincle^−/−^ to WT neutrophils in the blood without *E. coli* injection and in the peritoneal cavity (PC) after injection. *p < 0.05 relative to the control.

To determine whether Mincle regulates the infiltration of neutrophils from blood circulation into the peritoneal fluid in bacterial septic peritonitis, we conducted adoptive transfer experiments using a mouse peritonitis model (Fig. 6a). Purified Mincle^−/−^ BM neutrophils were labeled intracellularly with fluorescent 5-(and 6-) chloromethyl SNARF-1 acetate (SNARF-1, [red]), and WT neutrophils were labeled with the dye CFSE (green). A single-cell suspension (in a 1:1 ratio) was intravenously injected into recipient WT mice. Blood cells were harvested 2 h after the injection, and SNARF-1^+^ or CFSE^+^ cells were analyzed by flow cytometry. The ratio of transferred WT and Mincle^−/−^ neutrophils remained near 1:1 in the blood (Fig. 6a,c). Next, we intraperitoneally injected *E. coli* into the recipient mice and collected peritoneal cells 6 h after the injection. In contrast to the above result, Mincle^−/−^ neutrophils less efficiently migrated into the peritoneal cavity after *E. coli* injection compared with WT neutrophils (Fig. 6c). These data thus support the hypothesis that Mincle plays a role in regulating the recruitment of neutrophils into foci of infection during bacterial sepsis.

## Discussion

Death due to septic injury is directly linked with a significant proinflammatory innate immune response that leads to multiple organ failure. The successful resolution of an infection requires adequate pathogen recognition and the mounting of an immune response sufficient to kill the invading organisms and prevent their spread. Neutrophil recruitment is an initial event of the innate immune response and one of the most important factors in impeding pathogen multiplication. The mechanism regulating neutrophil migration during sepsis is poorly understood, however. Mincle, a major pattern recognition receptor, recognizes a variety of pathogens, such as *Mycobacterium tuberculosis*^21^,^38^, *C. albicans*^27^, *Malasezzia* species^26^, *Fonsecaea pedrosoi*^39^, as well as the endogenous ligand SAP130^25^, which is released from dead cells. In this study, we examined the role of Mincle as a key regulator of neutrophil infiltration in the pathophysiology of sepsis. Our results suggest that Mincle-mediated GRK2 regulation plays an important role in maintaining high expression of CXCR2 and improving neutrophil infiltration, thus reducing the mortality rate of sepsis.

CXCR2-dependent signaling plays a major role in neutrophil migration to bring these immune cells in contact with pathogens^40^,^41^. GRK2, a specific kinase that interacts with G-protein-coupled receptors, induces the phosphorylation of CXCR2, thereby modulating the CXCR2 signal^42^. Regulation of GRK2 expression, therefore, represents an effective means of regulating neutrophil migration. For example, the TLR4 signaling pathway modulates downregulation of GRK2, thereby decreasing CXCR2 desensitization and augmenting neutrophil migration^43^. In contrast to TLR4, TLR2 signaling negatively regulates CXCR2 surface expression by increasing GRK2 levels during severe sepsis^15^. Our study implicates Mincle as a promoter of neutrophil recruitment into foci of infection by enhancing CXCR2 signaling. Although we showed the transcriptional reduction of GRK2 by Mincle activation in this study, there is a possibility of post-translational regulation in Mincle-mediated GRK2 suppression. Our previous study demonstrated an association between MAPKs and Src family proteins in Mincle-mediated cell adhesion, providing some clues regarding the process of GRK2 suppression in septic peritonitis^24^. TDM-induced Mincle signaling activates MAPKs, and the Src family inhibitor prevents CD11b/CD18 upregulation in neutrophils. Interestingly, c-Src directly phosphorylates GRK2 to promote its proteasomal degradation, and p42/44 MAPK-mediated GRK2 phosphorylation also leads to GRK2 degradation^44^,^45^. Therefore, Mincle-mediated Src and MAPK activation may possibly trigger GRK2 degradation, thereby maintaining constant CXCR2 expression on the neutrophil surface. Further study is needed, however, to examine the mechanism of Mincle-GRK2 regulation.

Considering that bacterial infection and sepsis cause significant pathology due to host cell death via necrosis, apoptosis, and pyroptosis, as well as extracellular trap-associated neutrophil death^46^, it is conceivable that Mincle recognizes endogenous ligands that are released as “danger signals” from dead cells in the interaction between bacteria and host cells. Yamasaki *et al*. reported that Mincle recognizes a soluble factor released by necroptotic cells identified as spliceosome-associated protein 130 (SAP130), which is a component of the U2 small nuclear ribonucleoprotein (snRNP) localized in the nucleus of normal live cells^25^. In addition, Seifert *et al*. showed that the release of SAP130 by necroptotic cells promotes Mincle signaling, enhancing the progression of pancreatic ductal adenocarcinoma^47^. As both TDM-Mincle and SAP130-Mincle ligation display the same spleen tyrosine kinase-dependent inflammatory responses^21^,^25^, we used the well-known Mincle ligand, TDM, as a surrogate for SAP130. Consistent with the up-regulation of GRK2 in Mincle^−/−^ CLP mice, we identified reduced GRK2 expression by TDM stimulation in BM neutrophils. Therefore, SAP130 released from bacterial septic injury may promote neutrophil recruitment via Mincle signaling.

The number of circulating white blood cells and neutrophils are respectively 20% and 25% lower in Mincle^−/−^ mice compared with control C57BL/6 J mice (from the Mouse Phenome Database http://www.jax.org/phenome.^27^,^48^). Although the cell numbers found in the blood of Mincle^−/−^ mice in our current study were still within the normal range for C57BL mice, neutropenia could be a possible explanation for the high susceptibility to microbial sepsis of Mincle^−/−^ mice. However, we observed apparently normal extravasation of Mincle^−/−^ neutrophils in the thioglycolate peritonitis inflammation model (data not shown). In addition, Mincle deficiency did not affect LPS-induced mortality or cytokine production. Thus, it is unlikely that a correlation exists between the lower number of neutrophils circulating in the blood and the higher susceptibility of Mincle^−/−^ mice to septic injury.

A previous study investigated the development of pneumonic sepsis in Mincle^−/−^ mice^49^, and our results corroborate the finding that Mincle plays a protective role in host defense against infection caused by gram-negative bacteria. Mincle^−/−^ mice infected with *Klebsiella pneumoniae* exhibit increased bacterial burden and inflammatory cytokine levels that increase the risk of sepsis^49^, which is in agreement with our results shown in Mincle^−/−^ CLP and *E. coli*–induced sepsis mice. Sharma *et al*. identified a defect in NET formation in Mincle^−/−^ neutrophils that helps explain our data demonstrating impaired bactericidal activity against *E. coli* despite normal phagocytosis. However, we observed a defect in neutrophil infiltration in Mincle^−/−^ mice with either CLP-induced multibacterial infection or *E. coli* infection, whereas Sharma *et al*. found massive accumulation of neutrophils in the lungs of *K. pneumoniae*–infected Mincle^−/−^ mice. The discrepancy between our present results and those of Sharma *et al*. regarding neutrophil recruitment may be due to the different bacterial strains examined. Unlike the case of a single bacterial infection, CLP induces a polymicrobial infection of the peritoneum, with a localized focus. CLP also promotes necrosis of the cecum, which serves as a chronic source of inflammatory stimuli as the disease progresses^50^. Thus, the regulatory role of Mincle differs depending on the complexity of the infection. In addition, differences between *E. coli* and *K. pneumoniae* may contribute to the different symptoms. A previous study showed that pyogenic liver abscesses involving *E. coli* are more likely to be seen in older patients and lead to biliary disease and coexisting malignancy, whereas infections involving *K. pneumoniae* are more likely to occur in younger men^51^. Therefore, further studies will be necessary for clearer elucidation of the role of Mincle signaling in bacterial sepsis.

Our findings have shown that Mincle enhances neutrophil infiltration in CLP, *E. coli*-induced, and LPS-induced sepsis. Reduced neutrophil recruitment in CLP and *E. coli*–induced peritonitis of Mincle^−/−^ mice seem to be associated with their high mortality. However, similar survival rates were observed in Mincle-deficient and WT mice subjected to LPS-induced sepsis despite the former group having impaired neutrophil infiltration. Hence, considering that the number of recruited neutrophils by LPS-injection is roughly ten times lower compared to that of *E. coli* infection, it is plausible that LPS-mediated system inflammation does not strongly trigger neutrophil recruitment. Thus, while Mincle does indeed accelerate neutrophil infiltration, we propose that neutrophil infiltration is not a key factor for survival in LPS-induced endotoxic shock.

In our study, Mincle deficiency increases the susceptibility in *E. coli*-induced sepsis, but is dispensable in LPS-induced endotoxic shock. One possible explanation for this difference is that a number of outer membrane proteins on *E. coli* can activate neutrophil functions. For example, lipoprotein is one of the proteins on *E. coli* membrane and is recognized by TLR2^52^. We have already shown that co-activation of TLR2 and Mincle synergistically boost inflammatory responses in neutrophils^24^. In addition, we used live *E. coli* for infection, which likely produced more dead host cells resulting from fighting against the infection. These dead cells are one of the Mincle ligands, therefore, unlike LPS injection, live *E. coli* infection can activate Mincle-dependent signals.

In conclusion, our study provides evidence that Mincle plays a key role in mediating the pathophysiology of fulminant microbial sepsis. Specifically, Mincle appears to be necessary for neutrophil infiltration into the site of infection, in part, through upregulating CXCR2-mediated F-actin polymerization and chemotaxis. Our results also suggest that modulation of Mincle signaling may be a viable approach for treating sepsis.

## Materials and Methods

### Mice

Mincle^−/−^ mice (Clec4eMNA) were kindly provided by the Consortium for Functional Glycomics (http://www.functionalglycomics.org). The mice were back-crossed for over 10 generations to a C57BL/6 background. All mice were maintained in the specific pathogen–free facility of the Laboratory Animal Research Center at Yonsei University. Animals were maintained and procedures were performed with approval of the IACUCs of Yonsei University (permit number: IACUC-A-201509-463-02) in accordance to LABORATORY ANIMAL ACT of Korean Ministry of Food and Drug Safety for enhancing the ethics and reliability on animal testing through appropriate administration of laboratory animals and animal testing.

### Sepsis model

In the CLP model, mice were anesthetized by intraperitoneal (i.p.) injection with pentobarbital (50 mg/kg). After midline laparotomy, the cecum was exposed, mobilized, and ligated below the ileocecal valve, and punctured through both surfaces twice with a 22-gauge needle. For other sepsis models, mice received an i.p. injection of either a bacterial suspension containing 3 × 10^8^ live *E. coli* cells (strain DH5-α, Invitrogen) or lipopolysaccharide (LPS, 10 mg/kg; *E. coli* strain O55:B5, Sigma). For TDM-treatment in mice undergoing *E. coli*-induced peritonitis, TDM was prepared as a water-in-oil emulsion as shown previously^24^. Mice were intravenously injected with 100 μl emulsion containing 30 μg TDM (Sigma). 2 h after TDM treatment, mice were intraperitoneally injected with *E. coli*. Survival was recorded once daily for 10 d.

### Cytokine measurements

Cytokine concentrations (TNF-α and IL-6) were determined using Cytometric Bead Arrays (BD Biosciences) as per the manufacturer’s instructions. CXCL2 concentration was measured by an ELISA kit from R&D Systems in accordance to the manufacturer’s instructions.

### Bacterial counts

At 24 h after CLP, mice were anesthetized and peritoneal lavage fluid was collected. The peritoneal lavage fluid was serially diluted and cultured overnight on Mueller-Hinton agar (Difco Laboratories) at 37 °C. Peritoneal lavage fluid was also collected 24 h after the initiation of *E. coli*–induced peritonitis. Samples were serially diluted, plated on Luria-Bertani agar (Difco Laboratories), and incubated for 24 h at 37 °C. Bacteria were enumerated by counting the number of colony-forming units (CFU).

### Neutrophil influx

Peritoneal lavage fluid was collected at indicated times after CLP and initiation of *E.coli*–induced peritonitis. Total cell counts were determined using an automatic cell counter (Adam-MC, Digital Bio), and differential cell counts were performed on Cytospin slides stained with Diff-Quick. Results are expressed as number of neutrophils per cavity.

### Adoptive transfer of neutrophils

Neutrophil-depleted mice were prepared by i.p. injection of cyclophosphamide (Cytoxan; Bristol-Myers Squibb) (two injections on day 1 [150 mg/kg] and day 4 [100 mg/kg]), as previously described^53^. Bone marrow neutrophils were isolated from WT and Mincle^−/−^ mice and labeled with 5-(and 6-)carboxyfluorescein diacetate succinimidyl ester (CFSE; final concentration 5 μM) or seminaphthorhodafluor-1 (SNARF-1) acetate (final concentration 5 μM) at 37 °C for 10 min. Labeled cells were mixed (1:1) and then injected intravenously into WT neutropenic mice that had been challenged with 1 × 10^8^ CFU of *E. coli* for 2 h. The blood was collected 2 h after intravenous injection of the cell mixture without *E. coli* injection for before migration control. The peritoneal cavity was harvested 6 h after injection of the cell mixture. The number of adoptively transferred neutrophils from the blood and collected from the peritoneal cavity of recipient mice was determined using a FACS Calibur flow cytometer (BD Biosciences). Relative recruitment of WT and Mincle^−/−^ neutrophils was calculated as the ratio of the indicated populations in the peritoneal cavity.

### Flow Cytometry Analysis

Surface expression of CXCR2 was stained with anti-CXCR2 PE (242216, R&D Systems) antibody, and intracellular GRK2 was stained with primary rabbit anti-GRK2 (C-15, Santa Cruz Biotechnology) and with secondary FITC-conjugated goat antibody to rabbit IgG (Sigma) as per the manufacturer’s protocol. Cells were analyzed by FACS Calibur flow cytometer (BD Biosciences).

### Neutrophil isolation and *in vitro* neutrophil assay conditions

Mouse neutrophils from bone marrow were separated using a Percoll density gradient, as previously described^23^,^24^. Neutrophils were suspended in RPMI-1640 medium supplemented with 5% fetal calf serum.

Forty-eight-well microchambers (Neuro Probe) with a 5-μm pore polycarbonate membrane were used for chemotaxis assays. WT and Mincle^−/−^ bone marrow (BM) neutrophils were incubated with 5 μM calcein-acetoxymethyl ester (calcein-AM) at 37 °C for 30 min. Thereafter, the cells were washed, resuspended in RPMI containing 10% fetal calf serum, and allowed to migrate toward CXCL2 (10 ng/ml) or medium alone at 37 °C for 1 h. Non-migrating cells on the origin side of the membrane were removed by wiping with a tissue. Neutrophils that migrated through the membrane were identified based on the calcein fluorescence signal (excitation, 485 nm; emission, 530 nm).

For adhesion assays, calcein-AM–labeled WT and Mincle^−/−^ BM neutrophils were stimulated with CXCL2 (10 ng/ml) and incubated in wells coated with 1 μg/ml of fibronectin or fibrinogen. Control neutrophils were treated with medium lacking CXCL2. Cell adhesion was determined by measuring the fluorescence of adherent cells.

F-actin polymerization was assayed as described previously^24^. Briefly, WT and Mincle^−/−^ BM neutrophils were incubated with 10 ng/ml CXCL2 at 37 °C for 10 min. The cells were then fixed, permeabilized, and stained with fluorescein isothiocyanate–phalloidin (Molecular Probes, Eugene, OR). Images were captured using an Olympus DP-40 microscope, and the mean fluorescence intensity (MFI) was determined from a linear measurement of the fluorescence of individual cells. More than 20 cells from at least five randomly selected fields on each slide were analyzed.

### Immunoblot analysis

For immunoblotting, neutrophils were stimulated with LPS (100 ng/ml) and/or TDM (25 μg/ml) for 24 h at 37 °C. Then, cells were lysed with 2x Laemmli buffer and equal amounts of proteins were analyzed by immunoblot, and signals developed with Amersham ECL reagents were detected with the ImageQuant LAS 4000 system (GE Healthcare). Anti-GRK2 antibody (C-15) was obtained from Santa Cruz Biotechnology and anti-beta-actin antibody (#4967) was purchased from Cell signaling Technology. We used all antibodies in 1:1,000 dilution condition for immunoblot analysis.

### RNA extraction and quantitative RT-PCR

Total RNA was extracted by Trizol Reagent (Invitrogen) in accordance to the manufacturer’s protocol. cDNA was synthesized by Superscript II Reverse Transcriptase (Invitrogen) with oligo-dT as the primers. The expression of individual genes was measured by real-time PCR using a Bio-Rad CFX, and was quantitatively normalized to the housekeeping gene Gapdh by the change-in-cycling-threshold (ΔΔC_T_) method. The following primer sequences were used: 5′-GGC AAA TTC AAC GGC ACA GTC AAG-3′ and 5′-TCG CTC CTG GAA GAT GGT GAT GG-3′ for Gapdh; 5′-ATG CTG CCA CCT CTC TAC AAG-3′ and 5′-GGT CAC TTC TCC TCG GTC CT-3′ for GRK2; 5′-CAT CCC ACC CAC ACA GTG AAA GAG-3′ and 5′-CCT TCC ATG AAA GCC ATC CGA CTG-3′ for Cxcl2.

### RNA-Seq data analysis

RNA sequencing analysis was performed as previously described^23^. The GEO accession number for the RNA sequencing data reported in this paper is GSE70793.

### Statistical analysis

Statistical analysis was performed with Prism 6.0 software (GraphPad). An unpaired two-tailed *t-*test with 95% confidence interval was used for calculation of *P* values. For survival experiments, a log-rank (Mantel-Cox) test was used for calculation of *P* values. Group sizes, reproducibility and *P* values for each experiment are given in figure legends.

## Additional Information

**How to cite this article:** Lee, W.-B. *et al*. Mincle activation enhances neutrophil migration and resistance to polymicrobial septic peritonitis. *Sci. Rep.* **7**, 41106; doi: 10.1038/srep41106 (2017).

**Publisher's note:** Springer Nature remains neutral with regard to jurisdictional claims in published maps and institutional affiliations.

## Supplementary Material

Supplementary Information
