# Supplementary material for: Mincle activation enhances neutrophil migration and resistance to polymicrobial septic peritonitis
Source: Sci Rep. 2017 Jan 23;7:41106. doi: 10.1038/srep41106 (PMC5253726; doi:10.1038/srep41106)
Supplement: Supplementary Information [file srep41106-s1.pdf]

Supplementary information

Mincle activation enhances neutrophil migration and resistance to polymicrobial septic peritonitis

Wook-Bin Lee[1*], Ji-Jing Yan[2*], Ji-Seon Kang[1], Quanri Zhang[3], Won Young Choi[3], Lark Kyun Kim[4,5], Young-Joon Kim[1,3]

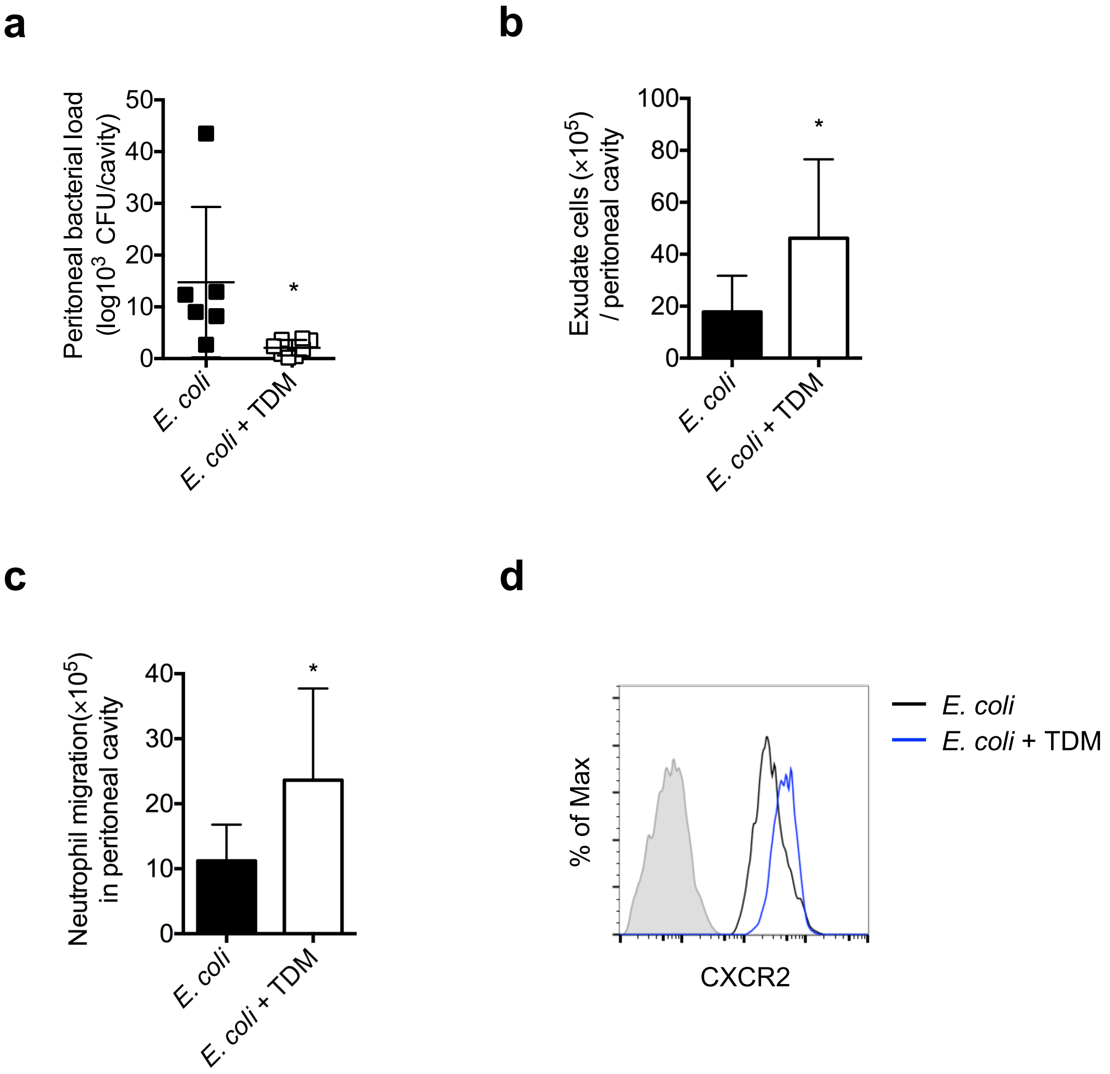

Supplementary Figure 1. TDM-treatment in mice alleviates *E. coli*–induced septic peritonitis. (**a**) Number of CFU in peritoneal exudate 6 h after *E. coli* injection or *E. coli* with TDM injection. Horizontal bars represent mean values, and squares represent individual mice (n=6 and 8, each). (**b**) Total cells and (**c**) neutrophils in peritoneal exudate of 6 h after *E. coli* or *E. coli* with TDM injection (n=8). Data are mean ± SD. *$p<0.05$ relative to *E. coli* injection. (**d**) Surface expression of CXCR2 on blood neutrophils measured using flow cytometry. Neutrophils (Ly6G+CD11b+) 6 h after *E. coli*–injected or *E. coli* with TDM-injected mice were measured.

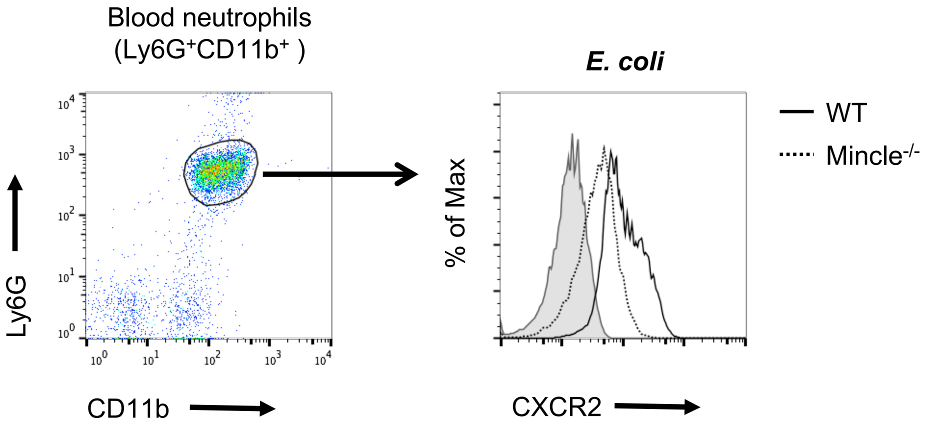

Supplementary Figure 2. Mincle$^{-/-}$ neutrophils show less CXCR2 surface expression compared with WT neutrophils during *E. coli*-induced peritonitis. Gating strategies for Ly6G^{+}CD11b^{+} blood neutrophils from 2 h after *E. coli*–injected WT and Mincle$^{-/-}$ mice. Surface expression of CXCR2 on Ly6G^{+}CD11b^{+} blood neutrophils measured using flow cytometry.
